# Factors influencing health-related quality of life in older adult women with sarcopenia: analysis of the Korean National Health and Nutrition Examination Survey 2019

**DOI:** 10.4069/kjwhn.2023.12.14

**Published:** 2023-12-28

**Authors:** Sol Hyun Lee, Ju-Hee Nho, Hye Young Kim, Eun Jee Lee

**Affiliations:** 1 Department of Nursing, Presbyterian Medical Center, Jeonju, Korea; 2 College of Nursing, Jeonbuk National University, Jeonju, Korea; 3 Research Institute of Nursing Science, Jeonbuk National University, Jeonju, Korea

**Keywords:** Aged, Quality of life, Sarcopenia, Women

## Abstract

**Purpose:**

This study aimed to identify factors influencing the health-related quality of life (HRQoL) of older adult women with sarcopenia.

**Methods:**

The study was secondary data analysis using data from the 2019 Korea National Health and Nutrition Examination Survey. The final sample consisted of 142 women aged 60 years and older with sarcopenia and were selected from 8,110 women. The participants was analyzed using complex descriptive statistics, independent t-test, and regression.

**Results:**

In terms of HRQoL, three general characteristics were found to be influential, with an explanatory power of 56.0%: difficulty climbing stairs, difficulty working, and perceived health status. Having no or mild difficulty when climbing stairs (B=.20, *p*=.001; B=.21, *p*<.001) and no or mild difficulty when working (B=.25, *p*=.002; B=.208, *p*=.013) had a significant effect on HRQoL compared to severe difficulty. Having good or ordinary perceived health status had a significant effect on the HRQoL (B=.11, *p*<.001; B=.09, *p*<.001).

**Conclusion:**

Based on study findings that the HRQoL of older adult women with sarcopenia were influenced by difficulty climbing stairs and working, as well as good perceived health status, healthcare providers should assist elderly women to maintain physical activities in their daily lives.

## Introduction

Sarcopenia is one of the diseases that degrade the health-related quality of life (HRQoL) of older adult women and the decrease in muscle mass due to aging is one of the representative changes [1]. Sarcopenia causes musculoskeletal diseases (e.g. falls and fractures), depression, or cognitive decline. In addition, chronic diseases such as heart failure and chronic obstructive pulmonary disease accelerate muscle loss, resulting in a vicious cycle. This not only has a great impact on the HRQoL but also has become an important public health problem [2].

The decrease in muscle strength and muscle mass begins at the age of about 40 years, and women in particular lose muscle mass at the same time they experience an increase in abdominal fat due to energy loss caused by aging and hormonal changes caused by menopause [3]. In an 8-year follow-up of a longitudinal study of aging with a sample of 3,404 people in the United Kingdom, women were at 20% higher risk of developing sarcopenia than men [4]. In particular, in Asians, women had a higher risk of sarcopenia due to higher body fat ratios than other ethnic groups and increased abdominal obesity [5]. Therefore, the management and prevention of sarcopenia for older Asian women diagnosed with sarcopenia is an important health problem.

Older adult women with sarcopenia have been reported as feeling lonely and depressed due to difficulties and social constraints in daily life related to aging and having suicidal thoughts, which can negatively affect their HRQoL [6]. In addition, a longitudinal study of 40–44 years-old participants reported that having sarcopenia at baseline was associated with worse scores of HRQoL at follow-up, compared to those without sarcopenia at baseline [7]. Another study of 4,937 Korean seniors aged 60 years or older found that HRQoL scores were significantly lower for sarcopenic women compared to their nonsarcopenic counterparts [8]. Moreover, as older adult women have a higher muscle reduction rate than older adult men and a longer life expectancy [9], they are a vulnerable group that is likely to be in a vicious cycle caused by sarcopenia. The quality of life (QoL) of older people with sarcopenia is also related to mental health, i.e., having sarcopenia was associated with a higher level of depression or anxiety, lower subjective health perception and nutritional status, and poorer QoL [10]. Moreover, because older people have many diseases, HRQoL was found to be related to the type of health insurance currently subscribed to and whether private medical insurance is available [11].

A useful model for identifying factors affecting HRQoL was modified by Ferrans et al. [12] from Wilson and Cleary’s HRQoL model [13]. This modified HRQoL model offers a framework that integrates the biomedical paradigm focused on the cause of disease and the social science paradigm focused on function and overall well-being. The model explains how HRQoL can be examined in terms of dynamic and multifaceted aspects while explaining the influence on the HRQoL through the following characteristics: individual characteristics, biological factors, symptoms, functional status, general health perceptions, and environmental characteristics [12]. Therefore, this study aimed to use the model to explore the factors influencing HRQoL in older women with sarcopenia.

Previous studies on sarcopenia in elderly women conducted in South Korea (hereafter, Korea) have focused on the effect of sarcopenic obesity on psychological health and QoL [6], the prevalence and factors related to sarcopenic obesity [14], and the prevalence of sarcopenia in association with activities of daily living, nutrition, and depression [14]. However, there are few studies that comprehensively sought to identify factors affecting the HRQoL based on Ferran’s HRQoL model [12] in older adult women with sarcopenia. Therefore, the current study was conducted to identify factors affecting HRQoL in older women with sarcopenia, to ultimately provide nursing evidence for improving their HRQoL.

This study aimed to identify factors influencing HRQoL of older adult women with sarcopenia based on Ferran’s HRQoL model [12], and the specific purposes are as follows.

(1) To identify the general characteristics of older adult women with sarcopenia

(2) To investigate the difference in HRQoL according to their general characteristics

(3) To determine the factors influencing their HRQoL

## Methods

**Ethics statement:** Obtaining informed consent was exempted by the Institutional Review Board of Jeonbuk National University (No. IRB-2022-08-021) because there was no sensitive information and the survey was anonymously treated.

### Research design

This study is a descriptive correlational study conducted to identify factors influencing HRQoL in older adult women with sarcopenia in Korea, analyzing the 8th Korea National Health and Nutrition Examination Survey (KNHANES) 2019 data. This study was described in accordance with the STROBE guidelines (https://www.strobe-statement.org/index.php?id=strobe-home).

### Data sources

**Figure 1.**
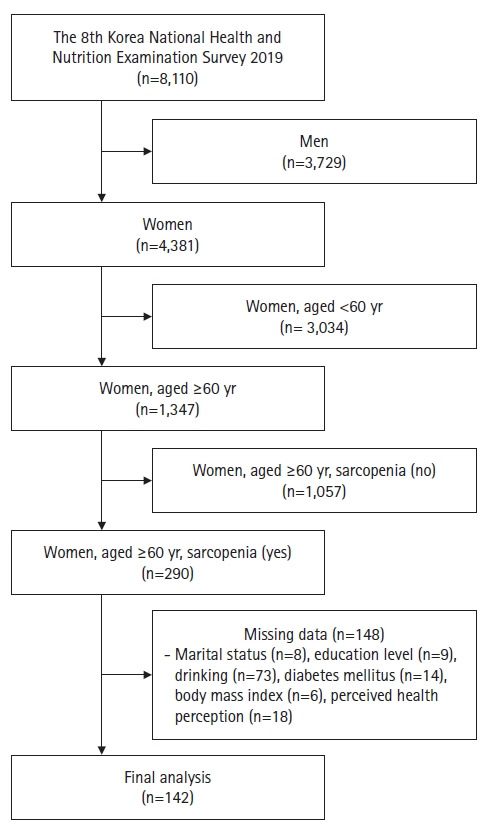
Flowchart of the study sample.

The 8th KNHANES was conducted in 2019 when the Korea Centers for Disease Control and Prevention conducted a survey of the annual National Health and Nutrition Survey with the approval of the Research Ethics Review Committee. Stratified sampling was done for all Koreans and from the 4,381 women out of 8,110 respondents of the KNHANES, 1,347 women aged 60 years or older were extracted. Subsequently, 290 elderly women with sarcopenia were again extracted. Excluding 148 persons with any missing information on even one of the variables considered in the study, the final analysis was done on a total of 142 elderly women with sarcopenia (Figure 1).

### Measurement

#### Sarcopenia

According to the Asian Working Group for Sarcopenia, sarcopenia is diagnosed based on handgrip strength (HGS), physical performance, and skeletal muscle mass, and a condition in which all three are reduced is classified as severe sarcopenia [2]. In the current study, sarcopenia is defined as HGS of less than 18 kg in women [2]. The maximum value of HGS of both hands or one hand measured three times was used.

#### Health-related quality of life

The HRQoL was analyzed using the EuroQol-5 Dimension (EQ-5D) instrument developed by the EuroQol Group [15]. The EQ-5D measures overall health and consists of five dimensions; mobility, self-care, usual activity, pain/discomfort, and anxiety/depression. Items are evaluated in three levels: ‘no problem,’ ‘moderate problem,’ and ‘serious problem’ and analyzed by the EQ-5D index, which is calculated from the prediction formula presented by the Korea Disease Control and Prevention Agency. A weight-adjusted value was calculated as 0 to 1 point and scores closer to 1 indicate better HRQoL.

#### Independent variables

Individual characteristics include age, marital status, income level, education level, employment, drinking status, and total sleep duration. Biological function includes disease and comorbidity. Symptoms include subjective perceptions and experiences, such as depression symptoms, suicidal ideation, and subjective stress. Functional status comprises physical, social, and role, such as climbing stairs and working. General health perceptions consist of subjective health evaluation, such as perceived health status. Environmental characteristics include health insurance, and interpersonal relationships, such as health insurance, private insurance, living area, and living type [12].

#### Individual characteristics

Age, marital status, education level, income level, employment, drinking, and sleep duration were included as the individual characteristics. Age was classified into early older adults aged 60 to 74 years and late older adults aged 75 years or older [16]. Marital status was classified into married or others, and education level was classified as elementary school or less, middle school, and high school or higher. Household income was classified into three groups according to the instructions for using KNHANES, lower (≤1 million Korean won [KRW]), middle (1–3 million KRW), and upper (>3 million KRW). Employment was classified into yes and no; drinking was classified into drinking and nondrinking; and total sleep duration was classified as 7 hours or more and less than 7 hours.

#### Biological function

Biological function includes osteoporosis, diabetes mellitus, body mass index (BMI), and waist circumference. Osteoporosis and diabetes were classified into yes and no, depending on whether they were diagnosed by a doctor. BMI was classified into <18.5 kg/m^2^ (underweight), 18.5–22.9 kg/m^2^ (normal), 23–24.9 kg/m^2^ (overweight), and ≥25 kg/m^2^ (obese). Waist circumference was classified by self-report as less than 85 cm (normal) and 85 cm or greater (obesity) [17].

#### Symptoms

Symptoms included depressive symptoms, suicidal ideation, and perceived stress. Depressive symptom was classified as yes and no for “depression for more than two consecutive weeks,” and suicide ideation were classified as yes and no for “serious suicide ideation over the past year.” Perceived stress was reclassified as “a lot (feeling a lot, feeling very much)” and “a little (feeling a little, feeling little)” about stress when asked about how much stress they felt in daily life.

#### Functional status

Functional status included difficulty climbing stairs and difficulty working during the past week. Difficulty of climbing stairs was classified as ‘no difficulty climbing stairs (no),’ ‘some difficulty climbing stairs (mild),’ ‘a lot of difficulty climbing stairs (severe),’ and ‘couldn’t climb stairs (very severe).’ Difficulty of working was classified as ‘no difficulty working (no),’ ‘some difficulty working (mild),’ ‘a lot of difficulty working (severe),’ and ‘couldn’t work (very severe).’

#### General health perceptions

General health perceptions were classified into good (very good, good), ordinary (normal), and poor (very bad, bad) based on the question, “How do you feel about your health in general?”

#### Environmental characteristics

Environmental characteristics included health insurance, private insurance, living area, and living type. Health insurance was classified into self-employed, employee, and dependent. The private insurance was classified into yes and no according to membership. The living area was classified into urban and rural, and the living type was classified into alone and together.

### Statistical analysis

The complex sample design was performed in consideration of the sample weight according to the sample design. Stratification variables and colony variables provided by the Korea Centers for Disease Control and Prevention were designated and analyzed. The data was analyzed using IBM SPSS ver. 26.0 (IBM Corp., Armonk, NY, USA), and significance set at *p*<.05. For participants’ characteristics, frequency and weighted percentage, estimated mean, and standard error (SE) were computed using complex sample frequency analysis. For the difference in HRQoL according to the general characteristics, independent t-tests were performed. Multiple regression analysis was performed for factors influencing HRQoL.

## Results

### Participants’ general characteristics

The mean age of the 142 participants was 72.77 years (SE, 0.57), and 51.3% were over 75 years of age. Overall, 72.3% of participants were unemployed and 54.2% were in the lower household income group. Regarding biological function, the most prevalent disease was osteoporosis (38.0%), followed by diabetes mellitus (27.5%). In terms of BMI, most respondents were normal (37.3%), followed by overweight (30.5%), and obesity (29.3%), and 55.7% were obese in waist circumference. Regarding symptoms, 16.1% of participants suffered from depression, 10.0% had experienced suicidal ideation, and 29.6% of participants reported a lot of stress in their daily lives. Regarding functional status, 47.9% and 42.2% of participants experienced mild difficulty climbing stairs and working, respectively, and 51.4% perceived their health status as ordinary. Regarding environmental characteristics, 62.9% of the employed and 58.3% of all participants did not have private insurance, 69.2% lived in urban areas, and 68.5% lived with someone (Table 1).

### Health-related quality of life according to the general characteristics of older adult women with sarcopenia

Married participants had significantly higher HRQoL than those whose status was otherwise (t=10.05, *p*=.002). In terms of symptoms, those who did not report depression symptoms and suicidal ideation had a higher HRQoL scare than those who did (t=5.02, *p*=.029; t=6.87, *p*=.011, respectively). Regarding functional status, those who had no or mild difficulty climbing stairs nor working had a higher HRQoL than their counterparts who had severe difficulty climbing stairs and working (F=8.72, *p*<.001; F=6.27, *p*=.001, respectively). In general, those with good or normal health perception showed a higher HRQoL than those with poor perception (F=14.32, *p*<.001). Regarding environmental characteristics, HRQoL was higher for those with private insurance (t=4.07, *p*=.048), and those living with someone (t=–3.65, *p*=.001) (Table 2).

### Factors influencing health-related quality of life of older adult women with sarcopenia

No and mild difficulty in climbing stairs were significantly associated with a higher HRQoL (B=.203, *p*=.001; B=.209, *p*<.001, respectively). In working, no and mild difficulty were also significantly associated with a higher HRQoL (B=.254, *p*=.002; B=.208, *p*=.013, respectively). Good or normal perceived health perceptions were significantly associated with a higher HRQoL (B=.111, *p*<.001; B=.087, *p*<.001, respectively). The explanatory power of these variables’ ability to explain HRQoL in older adult women with sarcopenia was approximately 56% (Table 3).

## Discussion

Regarding functional status, difficulty working had the greatest impact on the HRQoL of older adult women with sarcopenia. Working not only improves economic status but also self-esteem and QoL for older adult women with sarcopenia [18]. This may be because self-esteem increases through social ties and role performance; and through working people can feel less lonely or alienated, which may have a positive effect on subjective health awareness [19]. As the working-age population (15–64 years old) in Korea continues to decline and the older adult population rapidly increases, the possibility for older adult women to work may improve individual QoL and further contribute to sustainable growth in Korean society [20]. Therefore, community support systems that offer various social entry programs can help to create jobs for older adult women with sarcopenia. Follow-up studies that identify other related factors that affect the HRQoL of older adult women with sarcopenia, and intervention directions are also needed.

This study’s finding that HRQoL was higher with no difficulty climbing stairs is supported by a prior study that reported a statistically significant positive correlation between physical fitness variables, including stair climbing, with QoL in Korean low-income elders 65 years or older [21]. Another study [22] found that older people participating in a physical activity program including climbing stairs had more muscle strength. In addition to seeking to prevent and manage muscular dystrophy through exercise from middle age, when muscle mass begins to decrease [23] efforts to guide and educate older adult women with sarcopenia are needed, so that they can continue to practice climbing stairs in their daily lives.

Regarding general health perception, high HRQoL was associated with perceiving one’s health condition as good, which is consistent with other studies [4,24]. Perceived health perception is a comprehensive evaluation of one’s health in terms of physical, mental, social, and psychological aspects, and is reported to be closely related to depression and physical activity [25]. Given that health-promoting programs for older adults resulted in improving perceived health awareness and QoL [26], active management programs for older adult women with sarcopenia are needed.

A limitation of this study was that while sarcopenia should be studied in consideration of time changes to identify causal relationships, because cross-sectional KNHANES data that included HGS measurement were used, it was only possible to identify associated factors. Therefore, it would be beneficial to incorporate sarcopenia measures in future KNHANES data, e.g., physical performance or skeletal muscle mass, especially for high-risk groups. Also, while the revised QoL model describes the effect of individual and environmental characteristics on biological function and the interactions between an individual and their environment, this study did not list the results of these effects and interactions. Despite these limitations, this study is meaningful in that it applied Ferran’s QoL model [12] to identify the factors influencing the HRQoL of older adult women with sarcopenia.

In conclusion, this study’s findings show that it is necessary to actively implement supportive interventions that can reduce difficulties in daily life, such as working and climbing stairs for elderly women with sarcopenia. Findings can be applied by encouraging women to exercise from middle age, when sarcopenia can begin, and assisting older adult women to continue their physical activities. Finally, continuing and expanding sarcopenia measurement in national surveys and the development of interventions and health policies to improve the HRQoL of older adult women with sarcopenia are also needed.

## Figures and Tables

**Table 1.** General characteristics of participants (N=142)

Variable	Categories	n^†^ or Estimated mean±SE	Weighted (%)
Individual characteristics			
Age (year)		72.77±0.57	
	60–74	74	48.7
	≥75	68	51.3
Marital status	Married	65	44.5
	Others	77	55.5
Education level	≤Elementary school	80	54.1
	Middle school	50	38.3
	≥High school	12	7.6
Income	Lower	80	54.2
	Middle	31	23.9
	Upper	12	7.6
Employment	Yes	37	27.7
	No	105	72.3
Drinking	Yes	75	53.7
	No	67	46.3
Total sleep duration (hour)	≥7	73	50.7
	<7	69	49.3
Biological function			
Osteoporosis	Yes	58	38.0
	No	84	62.0
Diabetes mellitus	Yes	33	27.5
	No	109	72.5
Body mass index (kg/m^2^)	<18.5	5	3.0
	18.5–22.9	51	37.3
	23–24.9	43	30.5
	≥25	43	29.3
Waist circumference (cm)	≥85	80	55.7
	<85	62	44.3
Symptoms			
Depressive symptom	Yes	26	16.1
	No	116	83.9
Suicidal ideation	Yes	14	10.0
	No	128	90.0
Perceived stress	A lot	40	29.6
	A little	102	70.4
Functional status			
Difficulty climbing stairs	No	29	22.1
	Mild	69	47,9
	Severe	35	23.6
	Very severe	9	6.4
Difficulty working	No	41	31.0
	Mild	56	42.2
	Severe	33	18.4
	Very severe	12	8.4
General health perceptions			
Perceived health status	Poor	52	35.4
	Ordinary	72	51.4
	Good	18	13.2
Environmental characteristics			
Health insurance	Self-employed	41	26.5
	Employee	86	62.9
	Dependent	15	10.7
Private insurance	Yes	63	41.7
	No	79	58.3
Living area	Urban	94	69.2
	Rural	48	30.8
Living type	Alone	49	31.5
	Together	93	68.5

† Unweighted count (frequency).

**Table 2.** Quality of life according to general characteristics (N=142)

Variable	Categories	Mean±SE	F or t (*p*)
Individual characteristics			
Age (yr)	60–74	0.89±0.02	0.09 (.756)
	≥75	0.87±0.03	
Marital status	Married	0.93±0.02	10.05 (.002)
	Others	0.83±0.02	
Education level	≤Elementary school	0.87±0.02	.01 (.379)
	Middle school	0.86±0.04	
	≥High school	0.90±0.02	
Income	Lower	0.80±0.02	1.45 (.228)
	Middle	0.90±0.02	
	Upper	0.88±0.05	
Employment	Yes	0.90±0.02	2.64 (.109)
	No	0.86±0.02	
Drinking	Yes	0.88±0.02	0.04 (.838)
	No	0.88±0.03	
Total sleep duration (hour)	≥7	0.89±0.02	0.36 (.550)
	<7	0.87±0.02	
Biological function			
Osteoporosis	Yes	0.81±0.02	0.09 (.760)
	No	0.82±0.03	
Diabetes	Yes	0.77±0.04	3.43 (.069)
	No	0.86±0.01	
Body mass index (kg/m^2^)	<18.5	0.80±0.04	0.61 (.593)
	18.5–22.9	0.85±0.03	
	23–24.9	0.82±0.02	
	≥25	0.80±0.04	
Waist circumference (cm)	≥85	0.81±0.02	0.07 (.785)
	<85	0.82±0.03	
Symptoms			
Depressive symptom	Yes	0.74±0.04	5.02 (.029)
	No	0.83±0.02	
Suicidal ideation	Yes	0.72±0.01	6.87 (.011)
	No	0.85±0.01	
Perceived stress	A lot	0.81±0.03	1.39 (.242)
	A little	0.86±0.02	
Functional status			
Difficulty climbing stairs	No^a^	0.87±0.03	8.72 (<.001) (a,b>d)^†^
	Mild^b^	0.87±0.02	
	Severe^c^	0.72±0.03	
	Very severe^d^	0.64±0.06	
Difficulty working	No^a^	0.89±0.02	6.27 (.001) (a,b>d)^†^
	Mild^b^	0.83±0.02	
	Severe^c^	0.79±0.03	
	Very severe^d^	0.60±0.07	
General health perceptions			
Perceived health status	Poor^a^	0.65±0.04	14.32 (<.001) (c>b>a)^†^
	Ordinary^b^	0.83±0.03	
	Good^c^	0.87±0.03	
Environmental characteristics			
Health insurance	Self-employed	0.90±0.03	.32 (.724)
	Employee	0.89±0.02	
	Dependent	0.85±0.06	
Private insurance	Yes	0.91±0.03	4.07 (.048)
	No	0.85±0.03	
Living area	Urban	0.86±0.02	.59 (.446)
	Rural	0.90±0.04	
Living type	Alone	0.74±0.03	3.65 (.001)
	Together	0.89±0.01	

† Holm-Bonferroni method.

**Table 3.** Factors influencing quality of life of participants (N=142)

Variable	Categories	B	SE	t	*p*	95% CI
Individual characteristics						
Marital status^†^	Married	–.01	.02	–0.25	.803	–.05 to .04
Symptoms						
Depressive symptom^†^	No	.07	.04	1.82	.073	–.01 to .14
Suicidal ideation^†^	No	.04	.05	0.75	.456	–.06 to .14
Functional status						
Difficulty climbing stairs^†^	No	.20	.06	3.58	.001	.09 to .32
	Mild	.21	.05	4.03	<.001	.11 to .31
	Severe	.09	.06	1.59	.118	–.02 to .21
Difficulty working^†^	No	.25	.08	3.16	.002	.09 to .42
	Mild	.21	.08	2.56	.013	.05 to .37
	Severe	.18	.09	1.95	.056	–.01 to .36
General health perceptions						
Perceived health status^†^	Good	.11	.03	3.60	<.001	.03 to .05
	Ordinary	.09	.02	3.81	<.001	.02 to .04
Environment characteristics						
Private insurance^†^	Yes	.01	.02	0.36	.720	.04 to .13
Living type^†^	Together	.03	.03	0.86	.395	–.03 to .09
R^2^=.56, F=11.08, *p*<.05						

SE: standard error.

† Reference values were marital status (others), depression symptom (yes), suicide ideation (yes), difficulty climbing stairs (very severe), difficulty working (very severe), perceived health status (bad), private insurance (no), and living type (alone).
